# Effects of Microvesicles on Cell Apoptosis under Hypoxia

**DOI:** 10.1155/2019/5972152

**Published:** 2019-04-17

**Authors:** Ying Guo, Jin Tan, Yuyang Miao, Zuoming Sun, Qiang Zhang

**Affiliations:** ^1^Department of Geriatrics, Tianjin Medical University General Hospital, Tianjin Geriatrics Institute, Tianjin, China; ^2^Tianjin Medical University, Tianjin, China; ^3^Division of Immunology, Beckman Research Institute of the City of Hope, Duarte, CA 91010, USA

## Abstract

Hypoxia, as one of the severe cellular stresses, can cause cellular injury and even cell death. Apoptosis is the main mechanism of regulating cell death and is closely related to the cell death caused by hypoxia. However, hypoxia-induced apoptosis is not entirely the result of direct hypoxic stimulus of cells. In recent years, it has been found that cells injured by hypoxia can shed a kind of membranous vesicles, which are called microvesicles (MVs). MVs can carry bioactive molecules from injured mother cells and appear in blood, cerebrospinal fluid, and other body fluids. MVs can induce normal cell apoptosis by transferring bioactive molecules into adjacent cells and amplifying the hypoxic injury in an organism. This review summarizes the characteristic changes of MVs derived from hypoxic cells and the mechanism of normal cell apoptosis mediated by hypoxic cell-derived MVs. Finally, we introduce the significance of this apoptosis-apoptosis cascade reaction in hypoxic diseases.

## 1. Introduction

Hypoxia, as the main pathological mechanism of sleep apnea hypopnea syndrome, ischemic stroke, ischemic heart disease, and many other diseases, can cause endothelial cells, hippocampus neurons, and myocardial cells, as well as many other cells, injury and plays an important role in development and progression of disease [1–3]. Many studies have found that hypoxia mediates cell injury and even cell death mainly through oxidative stress, inflammation, acidosis, and apoptosis. Apoptosis, as the main mechanism of regulating cell death, plays a very crucial role in hypoxia-induced cellular injury [4].

Many results have found that there is a close relationship between hypoxia and apoptosis. Hypoxia can induce apoptosis by inducing mitochondrial damage, calcium overload, increased oxygen free radicals, increased expression of hypoxia-inducible factor (HIF), and so on. All along, most of the attentions have been focused on these common pathological mechanisms.

As new regulators of cell-cell communication, microvesicles (MVs) have received more and more attention in recent years. MVs are membranous vesicles with a diameter of 0.1-1 *μ*m and can be released by several types of cells when activated, injured, or undergoing apoptosis [5]. MVs can carry “cargo” from the cells from which they originate and transfer cargo into adjacent normal cells, thereby causing a series of changes in cells, including inducing cell apoptosis. It can be seen that cell injury is not entirely the result of direct stimulation of cells by hypoxia or other stimuli. It is also possible that injured cells indirectly act on peripheral normal cells by releasing the “mediator” (MVs), which contain substances that promote injury and apoptosis. It is the MVs that mediate the amplification of damage.

## 2. MVs and Hypoxia

### 2.1. MVs

**Figure 1:**
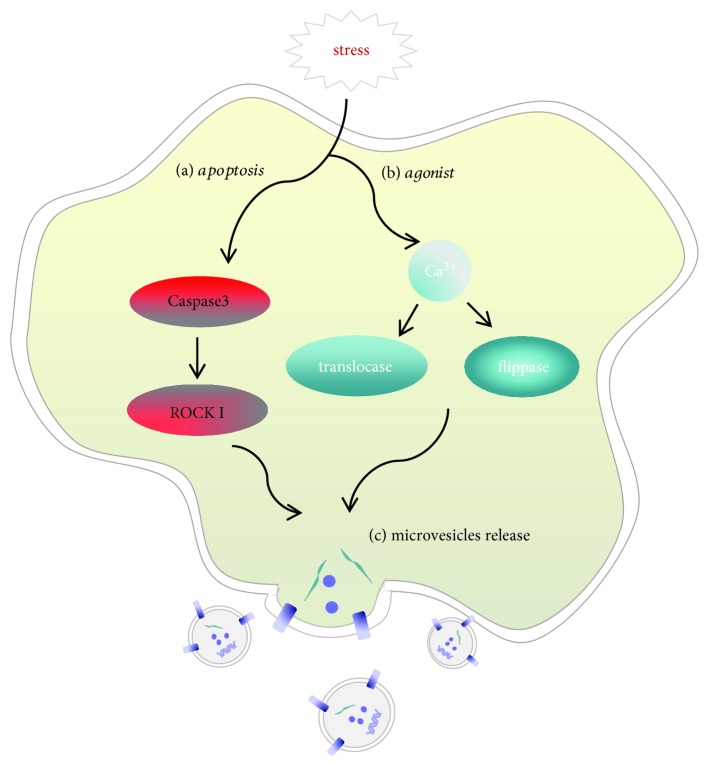
Differences in the release mechanism of MVs derived from activation or apoptosis of cells. (a) Apoptosis inducers activate caspase 3 and further activate ROCK I. (b) Activation inducers increase intracellular calcium and activate translocase and flippase. (c) Both of the mechanisms can lead to cytoskeleton destruction and MV release. The surface of MVs contains specific receptors, ligands, and other bioactivators from mother cells; it also contains DNA, miRNA, proteins, and other cargo.

Human cells undergo various stimuli such as activation and injury every day. When cells are exposed to these stimuli, they will shed small subcellular factors from the plasma membrane. However, it was not until the invention of the electron microscope in 1967 that the subcellular factors were proved to be derived from platelets and described as “platelet dust” [6]. With research development, the subcellular factors derived from many kinds of cells are called microvesicles. MVs are small membranous vesicles shed from the plasma membrane directly by budding, size in range from 0.1 to 1 *μ*m in diameter. Normal cells can release a small amount of MVs, but MVs are mainly derived from activated or apoptotic cells. The release mechanism of MVs during cell activation and apoptosis is not exactly the same. When cells are stimulated by activation inducers, the intracellular Ca^2+^ accumulates rapidly [7], and translocase and flippase are activated, while the activity of amino phospholipid translocase is inhibited; then, the negatively charged phosphatidyl serine (PS) flips over and exposes to the outer layer of cells [8]. When cells are stimulated by apoptosis inducers, cysteine aspartate protease 3 (caspase 3) can activate Rho-associated kinase (ROCK I), resulting in the contraction of actin and myosin [9]. Both activation and apoptosis can lead to membrane cytoskeletal disruption and MV generation [10] (Figure 1).

MVs do not have their own specific surface molecular markers but express surface markers derived from the cells they originate from. For instance, the surface of endothelial cell MVs (EMVs) can express endothelial cell surface adhesion molecule CD31. MVs derived from platelets can express the marker of activated platelets CD62P. MVs can also carry bioactive molecules such as DNA, signaling proteins, RNA, mRNA, and microRNA and mediate intercellular communication by transferring these bioactive molecules into surrounding cells. Therefore, MVs can be involved in a variety of physiological and pathological processes, such as autophagy, tumor growth and metastasis, cell apoptosis, and immune response [11–14].

### 2.2. Characteristic Changes of MV Release Induced by Hypoxia

Cells produce a small amount of MVs under normal conditions, but when cells are stimulated by hypoxia or oxidative stress, the production of MVs will increase significantly. Many studies have also shown that patients with hypoxic diseases have elevated levels of plasma MVs derived from multiple cell types. Nowadays, more and more studies have explored the role of MVs in the progression and diagnosis of hypoxic diseases.

#### 2.2.1. MVs Increased Significantly in Hypoxic Diseases

Stroke, including ischemic stroke, brain hemorrhage, and subarachnoid hemorrhage (SAH), can cause disturbed blood supply, ischemia, and hypoxia to the brain. The level of circulating MVs in stroke patients is related to a hypercoagulable state and poor clinical outcome. In brain hemorrhage, the plasma procoagulant MV level was significantly increased and was associated with stroke pathogenesis [15]. EMVs were significantly increased in SAH patients, the increased EMVs were related to symptomatic cerebral vasospasm, and platelet MVs (PMVs) might contribute to the pathogenesis of cerebral infarction attributable to vasospasm [16].

There is also a close relationship between MVs and ischemic cardiovascular disease. The levels of MVs derived from endothelial cells, platelets, leukocytes, and erythrocytes were significantly increased in patients with acute coronary syndrome (ACS) compared with healthy individuals and with patients with stable coronary disease [17, 18]. These phenomena confirmed the hypercoagulable state, as well as the activation of platelet and leukocyte observed in a clinical setting of ASC. Jung et al. showed that the levels of EMVs and PMVs in patients with ST segment elevation myocardial infarction (STEMI) were closely related to the size of myocardium at risk (MaR) and might reflect the severity of endothelial injury and platelet activation during myocardial infarction [19]. Circulating MVs play an important role in the progression and prognosis of ischemic cardiovascular disease by increasing endothelial injury, endothelial senescence, and thrombogenicity [20, 21]. In addition, MV levels in patients with different acute coronary occlusion patterns are also different. The level of EMVs was significantly increased in patients with sudden cardiac death (SCD) due to coronary artery occlusion compared with STEMI patients without rhythmic disturbances. In the future, EMVs may be a potential tool for prediction of the onset of arrhythmia and SCD due to coronary artery occlusion [22].

Obstructive sleep apnea hypopnea syndrome (OSAHS) is a kind of chronic sleep respiratory disease. Intermittent hypoxia (IH) caused by repeated upper airway obstruction is considered as the characteristic and the most important pathological and physiological pathways of OSAHS. OSAHS patients have been exposed to intermittent hypoxia for a long time, and the levels of EMVs, PMVs, and leukocyte MVs (LMVs) in their plasma are significantly increased, accompanied by endothelial injury and platelet aggregation [23, 24]. Moreover, Bikov et al. reported that the plasma CD41^+^ (platelet origin) MV level was directly correlated with the severity of OSAHS, and it might be a new marker for judging the severity of OSAHS in the future [25].

#### 2.2.2. Mechanism of Increasing MV Formation Induced by Hypoxia

Hypoxia-induced MV production is closely related to oxidative stress. Intermittent hypoxia induced an increase in EMVs of ApoE^−^/^−^ mice, and this increase could be blocked by antioxidant L-glutathione. In addition, an anti-inflammatory infliximab also significantly reduced the amount of circulating EMVs in mice [26]. It can be seen that oxidative stress and inflammatory response are related to EMV production under intermittent hypoxia. Teoh et al. also demonstrated that hypoxia/reoxygenation generated MVs from primary hepatocytes by processes that involved oxidative stress [27]. The mechanism of increasing MV formation induced by hypoxia is not clear, but some studies have shown that it may be related to hypoxia-inducible factor (HIF). HIF is a family of transcription factors necessary to maintain oxygen homeostasis and consists of two subunits, HIF-*α* and HIF-*β*. Studies have shown that hypoxia can increase the production of MVs derived from breast cancer cells by stimulating the expression of the HIF-dependent RAB22A gene [12]. In addition, hypoxic human trophoblasts increased MV production in human umbilical vein endothelial cells, and the proinflammatory protein HMGB1 (high-mobility group box 1) was a key factor in this process. Inhibition of HMGB1 in hypoxic trophoblasts could reduce the production of EMVs [28].

#### 2.2.3. Changes of MV Contents Induced by Hypoxia

MVs derived from hypoxic cells qualitatively differ from those derived from normoxic cells. Under hypoxic condition, MVs released by melanoma cells promoted the ability of adhesion and metastasis of melanoma cells. However, MVs released by normoxic melanoma cells did not have this effect [29]. This may be due to the fact that hypoxia changes MV contents so that hypoxic tumor MVs can play its own unique role. Similarly, it has been shown that hypoxic tumor-derived MVs inhibit more NK cell function as compared to normoxic tumor-derived MVs, and the mechanism is related to miR-23a packaged by hypoxic MVs [30]. Pulmonary embolism (PE) can cause hypoxia, hypercapnia, pulmonary hypertension, and other pathological changes. Circulating MVs in rats with PE showed significant elevation in proteins with prothrombotic characteristics, such as fibronectin precursor, fibrinogen alpha, and von Willebrand factor compared with MVs in control rats, and these proteins might be involved in the prothrombotic state in PE rats [31]. In brief, hypoxic stimulation can alter the levels and contents of MVs, allowing hypoxic MVs to exert their biological effects different from normoxic MVs.

## 3. Apoptosis Induced by MVs under Hypoxia

Under hypoxic conditions, MVs shed from injured cells and appear in a variety of body fluids. The process of mediating surrounding normal cell apoptosis by MVs can be divided into three steps. As extracellular substances, MVs should first bind to target cells; then, MVs transfer their contents by binding to target cells. Finally, these contents mediate normal cell apoptosis around the injured cells.

### 3.1. Combination of MVs with Target Cells

#### 3.1.1. Mechanism of Uptake of MVs by Target Cells

**Figure 2:**
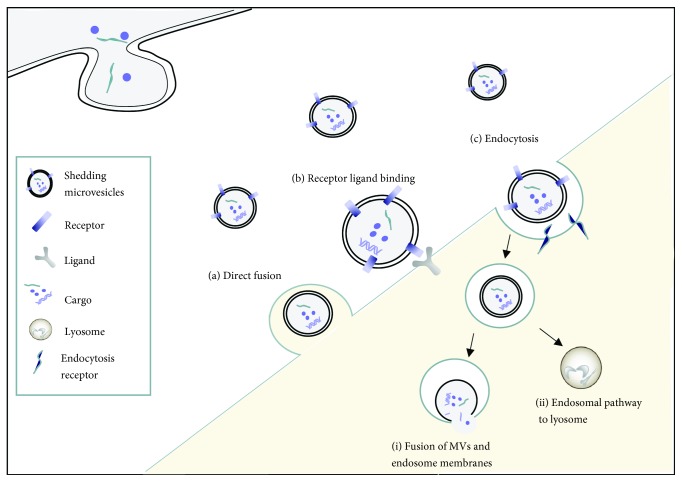
Combination of MVs with target cells. (a) Direct fusion: the MV membrane fuses with the target cell plasma membrane, releasing the cargo into the cytoplasm; (b) receptor ligand binding: specific receptors of MVs bind to ligands on the surface of target cells; (c) endocytosis: MVs are taken up by target cells through receptor-mediated endocytosis. The internationalization can be followed by (i) fusion of MVs and endosome membranes, then release of cargo and (ii) the endosomal pathway to lysosome: MVs targeting acidic lysosomes via the endosomal pathway.

The combination of MVs with target cells can be roughly divided into three types: receptor ligand binding, direct fusion, and endocytosis (Figure 2). Many cells can internalize MVs in a receptor ligand binding manner. For instance, monocyte-derived MVs could bind to activated platelets expressing P-selectin via P-selectin glycoprotein ligand 1 (PSGL-1) on their membrane [32]. Starvation-induced EMVs expressed Annexin I on their surface. At the same time, the corresponding phosphatidylserine receptor (PSR) was found on the surface of endothelial cells. It was Annexin I/PSR that mediated the uptake of EMVs by human coronary artery endothelial cells [33]. MVs derived from hypoxic bone marrow mesenchymal stem cells could be internalized by human umbilical vein endothelial cells (HUVECs). PS on the surface of hypoxic MVs and PSR on HUVECs were important participants in this internalization process [34]. MVs directly anchor the surface receptors of target cells through ligand on their surface, so as to transfer information between adjacent cells.

In addition, shedding MVs can also move some distance by diffusion and appear in body fluids, including blood, urine, and cerebrospinal fluid. They can be internalized by target cells through endocytosis or direct fusion. Endocytosis is one of the important ways in which target cells internalize MVs. For instance, PMPs could be taken up by active endocytosis in human brain endothelial cells (HBEC); the specific mechanism involved phagocytosis and macropinocytosis. Then, the internalized PMPs target the acidic lysosomes by the endosomal pathway [35].

#### 3.1.2. Cell Surface Molecules Regulate the Binding of MVs to Target Cells

The binding of MVs to target cells is also regulated by cell surface molecules. If the integrin A4 and CD29 on the surface of adult liver stem cell MVs were blocked with corresponding antibodies, it could inhibit the internalization of hepatic stem cell MVs in HepG2 hepatoma cells [36]. However, Wei et al. showed that CD29 and CD44 had no effect on the uptake of hypoxic stem cell MVs by HUVECs, but the addition of anti-PSR antibody could significantly block the uptake of hypoxic MVs by HUVECs. That is, PS on hypoxic stem cell MVs was the key molecule for the uptake of MVs by endothelial cells [34]. In addition, hypoxia could significantly promote the cellular internalization of MVs. Under hypoxic conditions, cultured peritubular endothelial cells (TEnCs) and tubular epithelial cells (TEpCs) could internalize endothelial progenitor cell MVs, and the level of internalized MVs was significantly higher than that of normoxic conditions. The blocking antibody assay showed that the adhesion molecule L-selectin was the main medium for internalization of MVs in hypoxic cells [37].

### 3.2. Release of Substances Carried by MVs

When MVs are taken up by target cells, they can release their contents into recipient cells. The contents that enter cells are the main participants in the process of apoptosis induced by MVs. MVs carry a wide variety of contents; even the newly discovered circular RNA (circRNAs), a novel noncoding RNA present in eukaryotic cells, has been found to be packaged and released in platelet-derived MVs. It is suggested that circRNAs may also participate in the communication between platelets and other cells through signaling pathways [38].

Among many substances carried by MVs, microRNAs are one of the most important participants in cell apoptosis. MicroRNA is a class of noncoding RNAs with a length of about 21-23 nucleotides. They can be transferred to the cytoplasm of target cells by MVs and then specifically bound to the 3′-terminal noncoding region of target mRNA, resulting in silencing of target gene expression. In recent years, it has been found that more and more microRNAs are involved in the regulation of apoptosis. PMVs caused apoptosis in lung cancer and colon cancer ectopic tumor by transporting miR-24. This platelet-derived miR-24 induced apoptosis by causing mitochondrial dysfunction [39]. Hepatic stem cell-derived MVs could transmit microRNAs with potential antitumor activity, such as miR-31, miR-223, and miR-451, into HepG2 hepatoma cells, thus inducing apoptosis of hepatoma cells [36].

### 3.3. Mechanism of Apoptosis Induced by MVs under Hypoxia

Many studies have shown that MVs can carry some independent proapoptotic signals, such as caspase 3 and activation of TRAIL. Abid et al. and Schneider et al. have demonstrated that MVs derived from HUVECs can carry caspase 3, and MVs induce apoptosis by transferring caspase into target cells [40, 41]. MVs derived from tumor cells could carry TRAIL to mediate T cell apoptosis [42]. In addition, specific ROS such as H_2_O_2_ or O^2-^ are also key mediators of apoptosis [43]. MVs could also carry ROS and transfer it to target cells to mediate cell apoptosis [44]. Furthermore, MVs affected cell survival by inducing intracellular oxidative stress [45]. The imbalance between proapoptosis protein Bax and antiapoptotic protein Bcl-2 mediated by MVs was also the key cause of cell apoptosis [46]. ElKeeb et al. showed that TF-rich MVs increased the expression of Bax and induced endothelial cell apoptosis through mechanisms mediated by P38 [47].

#### 3.3.1. P38 MAPK and JNK1/2 Signaling Pathways

Mitogen-activated protein kinase (MAPK) is a superfamily of serine/threonine protein kinase and is an important substance that transports signals from cytoplasm to the nucleus and causes nuclear changes. Among them, P38 MAPK and Jun-N-terminal kinase (JNK) are classical channels in the mammalian MAPK signaling pathway. It is believed that inflammation, ischemia/reperfusion, oxidative stress, and some other injuries can activate P38 MAPK and JNK pathways, thus regulating cell growth and differentiation, inflammation, apoptosis, and other pathological and physiological responses, and oxidative stress stimulation is especially important [48, 49]. Moreover, P38 MAPK can even affect intracellular oxidative stress in turn. Qiu et al. showed that lymphocyte-derived MVs activated P38 MAPK and increased intracellular oxidative stress. Excessive oxidative stress mediates caspase 3-dependent apoptosis [50]. Teoh et al. found that the inflammatory properties of MVs which were derived from mice ischemia/reperfusion injury were related to the activation of JNK. It was suggested that MVs and JNK were also closely related [27]. So, it is not surprising that MVs produced by hypoxic cells are closely related to P38 MAPK and JNK pathways.

There was direct evidence that MVs derived from HUVECs treated with hypoxia/reoxygenation (H/R) could carry more ROS than untreated HUVECs-MVs and transferred ROS to H9C2 cardiomyocytes. It also reduced the content of free radical scavenger SOD and increased the content of malondialdehyde (MDA), which further increased the ROS level in cells. Excessive oxidative stress in cells accelerated apoptosis by activating the p38 MAPK pathway and JNK1/2 signaling pathway. Moreover, H9C2 cells treated with HUVECs-MVs increased the activity of caspase 3, inhibited Bcl-2, and upregulated Bax, thus inducing apoptosis of H9C2 cardiomyocytes [51].

#### 3.3.2. PI3K/Akt Pathway

PI3K is a phosphatidylinositol kinase with lipid kinase and protein kinase activity. Serine/threonine kinase Akt is an important downstream target of the PI3K signal pathway; the PI3K/Akt pathway plays an important role in cell proliferation, apoptosis, and metabolism and is also the key pathway of ischemia/reperfusion [52, 53]. Endothelial nitric oxide synthase (eNOS) and NO are important downstream effectors of survival signal transduction during myocardial ischemia and reperfusion, and Akt can play a positive role in an ischemic heart by mediating eNOS phosphorylation and NO production [54].

Many studies have shown that MVs from hypoxic cells can affect the PI3K/Akt pathway. MVs derived from human microvascular endothelial cells reduced astrocyte proliferation by inhibiting the PI3K/Akt pathway [55]. MVs derived from the human pancreatic cancer cell line (HPC-4) increased anti-inflammatory cytokine production by monocytes through the PI3K/Akt pathway [56]. MVs isolated from hypoxic rat plasma significantly reduced the release of NO from aortic endothelial cells, and this effect was related to the reduction of phosphorylation of Akt on Ser473 kinase involved in eNOS phosphorylation [57].

Wang et al. demonstrated that MVs produced by endothelial progenitor cells (AEPC-MVs) carried high levels of caspase 3, transmitted them to H/R cerebral vascular endothelial cells (H/R HB-ECs), and further increased the number of ROS in cells. Meanwhile, the expression of eNOS and the production of NO were decreased, resulting in a significant increase in cell apoptosis. These harmful effects of AEPC-MVs could be alleviated by LY 294002, a PI3K inhibitor, suggesting that AEPC-MVs increase oxidative stress and apoptosis of H/R HB-ECs through the PI3K/Akt/eNOS pathway [58].

#### 3.3.3. Death Receptor Pathway

The death receptor (DR) belongs to the tumor necrosis factor receptor (TNFR) superfamily. The death receptor pathway is an exogenous apoptotic pathway. Currently, mammalian cell death receptors are mainly Fas, TNF receptors (TNFR), and TRAIL receptors (TRAILR), which can induce apoptosis by binding to the corresponding ligand FasL, TNF, or TRAIL on target cells.

FasL is a type II transmembrane protein originally described in immune cells; it can initiate the cascade of apoptosis of target cells after binding to the Fas receptor. MVs derived from human colorectal cancer cells carried FasL and TRAIL, induced T cell apoptosis in a dose-dependent manner after binding to Fas and TRAILR on the surface of activated T cells, and participated in the activation of downstream caspase. However, no further research has been done on exactly which caspase is activated [42].

Schock et al. showed that MVs isolated from plasma of chronic cerebral ischemia rats could induce apoptosis of rat renal cells. Apoptosis mediated by MVs was related to TNF-*α* and TRAILR, especially TNF-*α* receptor 1 and TRAIL receptor 4. The activation of TNF/TNFR and TRAIL/TRAILR pathways further activated caspase 3 and increased cell apoptosis. However, the addition of FasL antibody did not increase the survival rate of rat renal cells, indicating that this kind of MVs did not induce cell apoptosis by the Fas/FasL-dependent pathway. Unlike many other studies, Schock et al. did not see that MVs induced oxidative stress in rat renal cells. It might be related to different sources of MVs or different hypoxic conditions [59].

**Figure 3:**
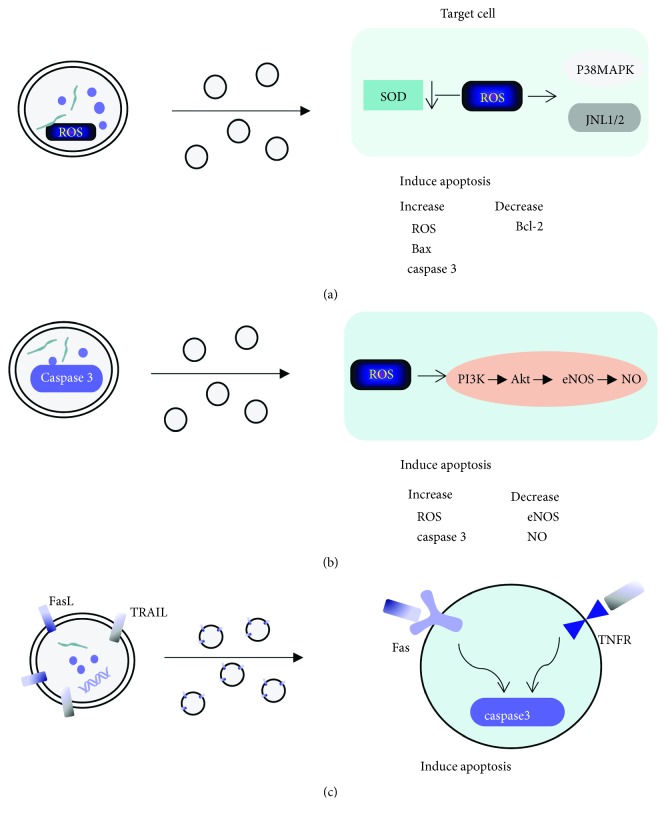
Different mechanisms of apoptosis induced by MVs. (a) MVs carry ROS and transfer it to target cells; increased oxidative stress in cells induce apoptosis through P38 and JNK1/2 pathways; (b) MVs carry caspase 3 and transfer it to target cells, increase the content of ROS in cells, and increase apoptosis by inhibiting the PI3K/Akt/eNOS pathway; (c) FasL and TRAIL on the surface of MVs bind to the corresponding receptors Fas and TNFR on the surface of target cells and participate in the activation of downstream apoptotic cascade reaction.

It can be seen that under hypoxic conditions, MVs released by injured cells mediate the related signal pathways through various types of contents, which affect the different stages of cell growth and development, thus mediating apoptosis of surrounding normal cells (Figure 3).

## 4. MVs Protect Cells against Apoptosis under Hypoxia

Generally speaking, it is believed that most of the time, MVs shed from the cell surface passively when cells are injured; so, they carry related harmful substances and mediate surrounding cell injury. Numerous studies have been surrounding the adverse effects of MVs released by injured cells. It does not mean that MVs can only mediate cell injury. In recent years, studies have found that MVs released by some special types of cells can also protect cells against apoptosis, especially the injury caused by hypoxia stimulation.

### 4.1. MVs from Stem Cells and Progenitor Cells

Progenitor cells, a circulating precursor of bone marrow, are adult stem cells that can locate at the site of damaged tissue and induce regeneration. Moreover, MVs derived from progenitor cells and stem cells can also play a protective role. MVs derived from bone marrow mesenchymal stem cells were rapidly internalized into injured renal tubules and glomeruli after injection into rats with renal ischemia/reperfusion. Internalized MVs played a protective role on acute renal injury by stimulating the proliferation and reducing apoptosis of renal tubular epithelial cells [60]. Endothelial progenitor-derived MVs carried microRNAs involved in cell proliferation, angiogenesis, and apoptosis inhibition, such as miR-126 and miR-296. By transferring these protective microRNAs, MVs protected hypoxic renal tubular endothelial cells and renal tubular epithelial cells from apoptosis, thereby protecting the kidney from acute ischemia/reperfusion injury [37]. Shedding MVs can carry the relevant substances from mother cells; this may explain the protective effect of MVs derived from stem cells and progenitor cells. MVs derived from induced pluripotent stem cells were rapidly taken up by cardiomyocytes. By reducing the activity of caspase 3, the oxidative stress injury induced by H_2_O_2_ and the cardiomyocyte apoptosis induced by ischemia/reperfusion injury were reduced. In addition, miR21 and miR210, which could protect myocardium from apoptosis, were also able to be transferred to H9C2 cardiomyocytes from induced pluripotent stem cell MVs, thus playing a role in myocardial protection [61].

MVs released by injured progenitor cells still have protective effects. Starvation-induced MVs produced by endothelial progenitor cells (sEPC-MVs) protected brain vascular endothelial cells from H/R-induced ROS and increased intracellular eNOS and NO production by activating the PI3K/Akt/eNOS pathway [58]. The reason why characteristics of mother cells exceed the effects of adverse stimulation on shedding MVs is not clear. This phenomenon may be related to the types of adverse conditions, but more detailed reasons need to be further explored.

### 4.2. Other Types of MVs Protect Cells against Apoptosis

MVs that have protective effects on cells are not only derived from stem cells and progenitor cells. Wang et al. showed that coincubation of ischemic myocardium-derived MVs with ischemia/reperfusion cardiomyocytes significantly reduced cardiomyocyte apoptosis and decreased the activity of caspase 3, caspase 9, and caspase 12. The expression of Bax decreased, and the expression of Bcl-2 increased. In addition, the expressions of glucose-regulated protein 78 (GRP78), sarcoplasmic/endoplasmic reticulum Ca^2+^-ATPase 2 (SERCA2), and phosphorylated phospholamban (p-PLB) increased, suggesting that MVs derived from ischemic myocardium could reduce cardiomyocyte apoptosis through mitochondrial and endoplasmic reticulum pathways [62].

## 5. Significance of Apoptosis Regulated by MVs in Hypoxic Diseases

### 5.1. Significance for Hypoxic Tumor Disease

Necrosis and hypoxia are the most common conditions in microenvironment of solid tumors, which are related to a fact that the existing blood vessels cannot meet the increasing demand for oxygen in rapidly expanding tumors, and hypoxia is closely related to the prognosis of tumors. As an important mediator derived from hypoxic tumor cells, MV has an intriguing influence on tumor progression and prognosis (Table 1).

On the one hand, MVs can promote the proliferation of tumor cells. MVs released by glioblastoma tumor cells promoted human glioma cell growth by transporting RNA and proteins [63]. On the other hand, MVs can promote tumor angiogenesis. For instance, hypoxia (1% O_2_) induced the release of human lung cancer cell MVs. These MVs stimulated the expression of a variety of cytokines in stromal cells to promote angiogenesis and improved the hypoxic state of tumor microenvironment [64]. In addition, immune escape mediated by MVs also plays an important role in tumor progression. MVs derived from human colon and rectal cancer cells transported miR-126 and TGF-*β* to NK cells and played an immunosuppressive role by inhibiting the effect of NK cells [30]. Colorectal cancer cells induced T cell apoptosis by releasing MVs containing FasL and apoptosis-inducing ligands [42]. Melanoma cells released MVs containing FasL and mediated T cell apoptosis through the Fas/FasL pathway. It can be seen that MVs prevent lymphocytes or other immunocytes from playing an antitumor role by inducing apoptosis and then promote tumor progression [65]. Besides, tumor-associated thrombotic events often lead to temporary vascular obstruction and acute hypoxia, which are also the main causes of prognosis deterioration in tumor patients. In fact, many studies have shown that tumor-derived MVs can promote coagulation. Human pancreatic cancer cells released tissue factor-bearing MVs which were closely related to thrombosis [66]. Moreover, some studies found that tumor-derived MVs could induce muscle cell apoptosis by binding their content miR-21 to the Toll-like 7 receptor on muscle cells, resulting in loss of skeletal muscle mass [67]. Therefore, the apoptosis induced by MVs may be in part associated with the progression and prognosis of solid tumors.

However, not all apoptosis induced by MVs have adverse effects on hypoxic diseases. PMVs suppressed tumor growth by inducing apoptosis of lung and colon carcinoma ectopic tumors [39]. MVs derived from hepatic stem cells induced apoptosis by transferring miRNAs with potential antitumor activity into hepatoma cells [36]. The intriguing role of tumor MVs may provide some new insights into the therapeutic intervention of tumors in the near future.

### 5.2. Significance for Ischemic Heart Disease

The pathological process leading to ischemic heart disease (IHD) is very complicated, including myocardial infarction, angina pectoris, or both, with ischemia/reperfusion injury (IRI) [68]. It is widely believed that oxidative stress is an important cause of IRI injury. Endothelial progenitor cell-derived MVs could reduce the increase of the ROS level induced by H/R and then protect cardiomyocytes [58]. In addition, induced pluripotent stem cells inhibited the expression of caspase 3 and protected ischemic cardiomyocytes from apoptosis [61].

Remote ischemic preconditioning (RIPC) before myocardial reperfusion is a protective strategy in acute myocardial infarction. Giricz et al. demonstrated that MVs released from an ischemic preconditioning heart reduced myocardial infarction size in ischemia/reperfusion injury rats [69]. MVs derived from myocardial ischemic rats reduced apoptosis of cardiomyocytes in ischemia/reperfusion injury through mitochondrial and endoplasmic reticulum pathways, demonstrating the importance of MVs in IHD [62].

## 6. Summary and Future Perspectives

In this review, we focus on the effects of hypoxia on MV release. Increased release of different MVs can be observed in hypoxic diseases, and MVs play an important role in the pathogenesis of hypoxic diseases. Nowadays, people have studied more about the molecular specificity of MVs derived from different cell types. It will help people understand the physiological and pathological effects of MVs and use them as diagnostic tools for diseases. MVs may also become special markers for predicting disease progression and prognosis in the near future.

MVs play an important role in development and progression of hypoxic diseases such as IHD, ischemic stroke, OSA, and tumor. Many previous studies have focused on the harmful effects of MVs, such as promoting inflammation and apoptosis. However, in recent years, there is more and more evidence that MVs can also play a protective role, and protective MVs are not limited to stem cells and progenitor cells.

MVs are small in diameter, low in immunogenicity, and can carry proteins, RNA, and other substances through the biomembrane to mediate information exchange with target cells. These advantages suggest that MVs may become a new therapeutic intervention as drug carriers in the future, although how they directly target specific cell targets remains to be further studied. In addition, most of the studies focus on cell and animal experiments; clinical research is relatively less. However, it still does not hinder the potential of MVs to become new therapeutic drugs.

## Figures and Tables

**Table 1:** The role of MV in the tumor.

Main role	MV origins	Acting cells	Type of action
*Promote tumor progression*			
Promote growth	Glioblastoma cells	Human glioma cells	MVs transport RNA and proteins
Promote tumor angiogenesis	Human lung cancer cells	Stromal cells	Express more angiogenic factors
Mediated immune escape	Human colorectal cancer cells	NK cells	MVs transport miR-126 and TGF-*β*
	Melanoma cells	T cells	MVs transport FasL
Promote thrombosis	Human pancreatic cancer cells	Platelets	MVs transport tissue factor
Promoting normal cell apoptosis	Lung and pancreatic tumor cells	Myoblasts	MVs transport miR-21
*Inhibited tumor progression*			
Induce tumor cell apoptosis	Platelets	Lung and colon carcinoma ectopic tumors	MVs transport miR-24
	Human hepatic stem cells	Hepatoma cells	MVs transfer miRNAs with potential antitumor activity
